# Spatial Optimization of Electrophysiological Signal Acquisition in *Clivia* Leaves Under a Controlled Leaf-Surface Salt-Treatment Model

**DOI:** 10.3390/plants15091363

**Published:** 2026-04-29

**Authors:** Ji Qi, Yuchao Yang, Yicheng Wang, Haoran Wang, Qiuping Wang, Yan Shi, Yanwei Wang, Hong Men

**Affiliations:** 1 School of Automation Engineering, Northeast Electric Power University, Jilin 132012, China; 2 College of Mechatronics, Changchun Polytechnic University, Changchun 130033, China; 3 Institute of Advanced Sensor Technology, Northeast Electric Power University, Jilin 132012, China

**Keywords:** *Clivia*, plant electrophysiology, leaf-surface salt treatment, spatial heterogeneity, signal acquisition optimization, lightweight deep learning

## Abstract

Plant electrophysiological signals can rapidly reflect the dynamic responses of plants to external stimuli, giving them strong potential for nondestructive monitoring and early state recognition. However, differences among plant organs, as well as spatial heterogeneity within the same organ, may substantially affect signal quality and stability because of variations in tissue structure and local physiological activity. To address this issue, this study used *Clivia* as an experimental model and established a controlled local leaf-surface salt-treatment paradigm to systematically evaluate the relative discriminative ability of electrophysiological signals recorded from different spatial positions on leaves. First, stepwise screening of longitudinal leaf regions and leaf hierarchy was performed using 0 mM and 100 mM NaCl agarose gel treatments to determine the optimal signal acquisition position. Then, based on the selected position, a five-level NaCl treatment recognition task was constructed, and LRPNet, a residual network integrating PoolFormer and an efficient channel attention mechanism, was proposed for multi-gradient classification of plant electrophysiological signals. The results showed that, within the current experimental framework, the basal region of the top leaf exhibited the highest relative separability and the best overall recognition performance. In the five-gradient recognition task, LRPNet achieved the highest mean Accuracy of 92.21% among the compared models. These findings indicated that plant electrophysiological signals exhibited pronounced spatial heterogeneity and that optimization of the recording location was not merely an experimental detail, but an important upstream factor that affected downstream recognition performance. This study provides a methodological basis for optimizing signal acquisition positions and improving electrophysiological signal recognition in plants. However, the present conclusions are mainly applicable to the controlled local salt-treatment paradigm established in this study and still require further validation through more rigorous physiological verification, cross-scenario testing, and more independent data-partitioning strategies.

## 1. Introduction

Plants do not passively endure environmental fluctuations. Instead, they perceive and integrate external information through multiple signaling pathways, including chemical, mechanical, hydraulic, and electrical signals, and then rapidly adjust growth, metabolism, and defense processes accordingly [1,2,3]. In recent years, research on the plant electrome has shown that the continuously changing bioelectrical activity within plants is not unstructured noise. Rather, it is a dynamic information carrier that is closely associated with environmental perception, changes in tissue status, and systemic responses [1,2]. Compared with morphological phenotypes, end-point biochemical indicators, or delayed visible symptoms, plant electrophysiological signals respond rapidly, can be recorded continuously, and provide high temporal resolution. They are therefore considered a promising entry point for early plant state monitoring and nondestructive sensing [1,2,3,4].

Mechanistically, the generation and propagation of plant electrical signals depend on changes in membrane potential, ion fluxes, hydraulic disturbances, and coupled transmission across cells and tissues [2,3]. Recent reviews have indicated that action potentials, slow wave potentials, and other local-to-systemic electrical responses together form a rapid information network through which plants respond to external stimuli. These signals are often accompanied by downstream changes in Ca^2+^, pH, reactive oxygen species, and osmotic status [1,2]. Li et al. further proposed that plant electrical signals can be regarded as a form of “plant electrophysiological phenotype,” whose central value lies in compressing complex physiological processes into continuously observable time-series representations [3]. This view suggests that, if high-quality signals can be stably acquired from appropriate locations, plant electrophysiology may serve as an important bridge between physiological mechanisms and intelligent recognition algorithms.

However, electrophysiological recording in plants is highly sensitive to the acquisition interface and the recording environment. Previous studies have shown that plant surface electrical signals usually have low amplitudes and strong nonstationarity. They are also easily affected by electrode contact impedance, leaf surface wetness, plant micromovements, electromagnetic interference, and fluctuations in temperature and humidity [1,3,5,6,7]. In unshielded environments, external noise may further increase recording complexity [8]. Studies on flexible and wearable plant sensors have also shown that the leaf surface is not an ideal rigid measurement plane. Epidermal microstructure, stomatal distribution, cuticle condition, and local water dynamics can all influence interface stability and signal repeatability [5,6,7,9]. Therefore, for plant electrophysiological recognition tasks, the acquisition location is not a negligible experimental detail. Instead, it is a key upstream variable that directly constrains signal quality and the upper limit of model performance.

In salt-related stimulation studies, two distinct scenarios should be clearly separated. One is long-term rhizosphere salinization, which is more common in agricultural production. The other is controlled local salt treatment on the leaf surface under laboratory conditions. Soil salinity usually affects osmotic potential, ionic homeostasis, and nutrient uptake in the root zone first, and then extends to the entire plant [10]. At the same time, foliar salt exposure is not entirely detached from real-world conditions. In coastal regions, sea-salt spray, salt fog, and aerosol deposition, as well as deliquescent salt particles that form liquid films under high humidity, can directly expose leaf surfaces to saline environments. Foliar solute uptake may also occur under such conditions. Ossola et al. noted that the leaf surface is not an inert boundary. Instead, it is a “chemical landscape” that is closely coupled with atmospheric particles, salt deposition, and droplet reactions [9]. Barlas et al. further demonstrated that leaf wettability and salt hygroscopicity strongly influence foliar absorption, and that different salts and leaf surface properties can lead to markedly different foliar input behaviors [11]. Therefore, although local foliar salt treatment cannot be equated with long-term root-zone salt stress, it can still provide a controlled model with clear comparative boundaries. Within this model, investigating how different spatial positions on leaves respond to salt-induced electrophysiological changes has clear methodological value. It can help identify more suitable recording regions and clarify the relationship between spatial heterogeneity in plant electrical signals and recognition performance.

Substantial progress has also been made in signal acquisition and plant–sensor interface engineering. Reviews by Yan et al. and Wu et al. have shown that wearable and flexible sensors enable continuous and minimally invasive recording on leaf surfaces. These studies also emphasized that adhesion, breathability, and mechanical compatibility at the plant–sensor interface are critical for long-term measurement stability [5,6,7]. Zhou et al. further developed a microneedle-array-based plant electrophysiological sensor that enabled electrical signal mapping and recognition of plant state changes [4]. Collectively, these studies indicate that plant electrophysiological monitoring is moving beyond the question of whether signals can be recorded. The field has increasingly focused on achieving stable signal recording, improving information quality, and enabling intelligent recognition. However, most existing studies have concentrated on electrode materials, signal denoising, or network design, with comparatively few systematically examining how different spatial acquisition locations within the same plant influence signal separability and model performance. For plant electrophysiological data, which are typically low in amplitude and weak in stability, the choice of recording location may itself be a decisive upstream step that determines the upper bound of the final results.

The integration of plant electrophysiology with machine learning and deep learning has also advanced considerably in signal analysis and pattern recognition. Yao et al. used deep learning to predict long-term salt-related plant electrical signals and distinguish differences in salt tolerance, which suggests that plant electrical signals contain learnable structure [12]. González I Juclà et al. achieved early deep-learning-based recognition of nitrogen deficiency in tomato under greenhouse conditions using raw electrophysiological data [13]. Yeom et al. and de Toledo et al. further showed that plant electrical signals can be used not only for binary detection but also for discriminating different environmental stimuli, including temperature, light, and water conditions [14,15]. These studies support the view that plant electrical signals contain temporal structures that are useful for pattern recognition. However, most existing work has emphasized feature engineering or network architecture design while leaving a more fundamental question insufficiently addressed: from which spatial position should signals be acquired so that the model receives the most discriminative responses?

Against this background, this study was organized around three sequential objectives: optimization of spatial acquisition location, validation of the optimal location, and construction of a lightweight recognition model. Using *Clivia* as the study species, a controlled local foliar salt-treatment model was established. The electrophysiological separability of different longitudinal leaf regions and different leaf hierarchies was first compared to identify the optimal recording location. Based on this optimal location, a five-gradient salt-treatment recognition task was then constructed, and LRPNet, a lightweight residual network integrating PoolFormer and the Efficient Channel Attention (ECA) mechanism, was developed to improve recognition performance for multi-gradient plant electrophysiological signals. The aim of this study is not to directly equate local foliar salt treatment with long-term root-zone salt stress. Rather, it is to systematically characterize the relationship among spatial position, signal quality, and recognition performance within a controlled model, and to provide a basis for optimizing plant electrophysiological acquisition strategies and designing lightweight recognition methods.

The main contributions of this study are summarized as follows:

(1) A multi-location electrophysiological dataset for *Clivia* under controlled local foliar salt treatment was constructed, and a unified experimental framework spanning spatial position screening and five-gradient recognition was established.

(2) Under a unified comparison framework, we systematically evaluated the relative separability of different longitudinal leaf regions and different leaf hierarchies, and identified the basal region of the top leaf as the optimal signal acquisition location in the current experimental model.

(3) We proposed the lightweight residual network LRPNet, which achieved the highest mean performance among the compared models on the five-gradient recognition task at the optimal acquisition location.

## 2. Results and Discussion

### 2.1. Analysis of Pre-Processing Results

**Figure 1:**
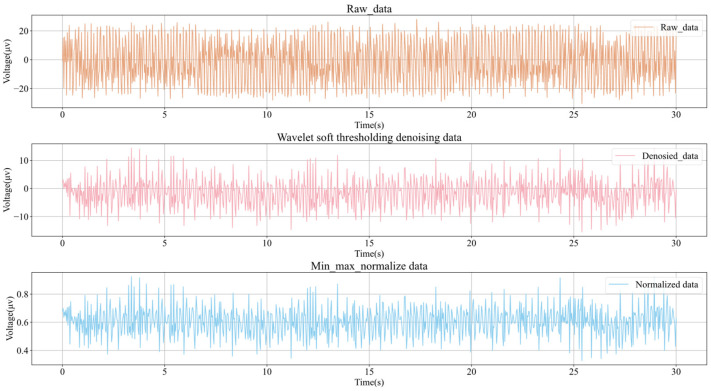
Example of preprocessing for a single electrophysiological sample recorded from Clivia under the 0 mM NaCl treatment.

As shown in Figure 1, the raw electrophysiological waveforms under different salt-treatment gradients all exhibited pronounced random fluctuations and a certain degree of baseline variation. This observation is consistent with the known characteristics of plant leaf-surface electrical signals, which are low in amplitude, non-stationary, and sensitive to environmental conditions [1,3,8,12,16,17]. Visual inspection showed that changes in treatment concentration were associated with differences in both waveform center position and local fluctuation envelope, suggesting that treatment intensity may influence electrophysiological responses at both the temporal-pattern level and the absolute-potential level.

After wavelet threshold denoising, high-frequency random interference was effectively suppressed, while the low-frequency main trend and locally meaningful fluctuations were preserved. Previous studies have shown that, for low-signal-to-noise and nonstationary time series such as plant leaf-surface electrical signals, appropriate denoising can improve the identifiability of major temporal patterns and reduce the influence of environmental noise on subsequent feature extraction and classification training [16,17]. On this basis, normalization further reduced differences in amplitude scale among samples, allowing model training to focus more on waveform structure and dynamic patterns rather than numerical magnitude differences across samples.

Overall, the denoised and normalized signals retained the main variation trends while showing greater consistency and comparability, making them suitable for subsequent spatial sensitivity analysis and multi-gradient classification. However, normalization also weakened, to some extent, the information contained in the absolute amplitude and baseline offset of the raw signals. Therefore, the subsequent results in this study mainly reflected the separability of normalized electrophysiological time series at the waveform-pattern level under different treatment conditions. In other words, the current pre-processing pipeline was better suited to determining whether electrophysiological waveform structures showed stable and recognizable differences under different treatment conditions, whereas the independent contribution of absolute potential magnitude to recognition performance still requires further investigation.

### 2.2. Classification Results for Different Longitudinal Leaf Regions

To validate the proposed framework, six representative deep learning architectures were selected as baseline models, including the classical convolutional networks AlexNet [18] and VGG16 [19], the deep residual network ResNet-18 [20], the lightweight networks MobileNetV2 [21] and ShuffleNetV2 [22], and the representative vision Transformer model ViT-Base [23]. These models cover multiple architectural paradigms, including conventional convolution, residual learning, lightweight mobile-oriented design, and global self-attention modeling. This comparison therefore provides a structured basis for evaluating the relative advantages of both the optimal acquisition location and LRPNet.

**Figure 2:**
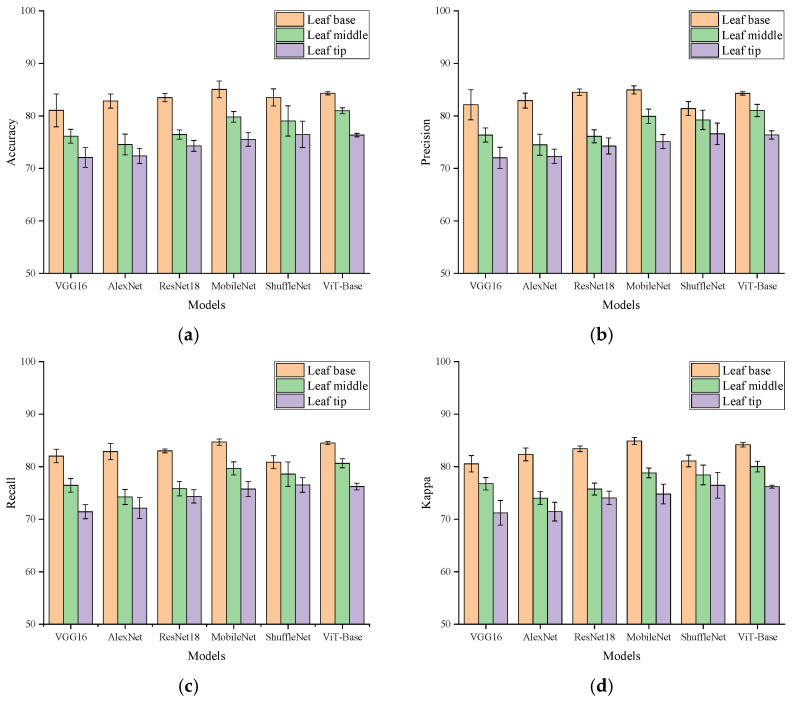
Comparison of multi-model classification performance across the three longitudinal leaf-region datasets. (**a**) Accuracy; (**b**) Precision; (**c**) Recall; (**d**) Kappa. Error bars represent the mean ± standard deviation across five repeated runs.

As shown in Figure 2, the multi-model comparison revealed a highly consistent ranking pattern across the three longitudinal leaf-region datasets. For all six models, Accuracy, Precision, Recall, and Kappa were generally higher for the basal-region dataset than for the middle- and apical-region datasets. This result indicated that, under the controlled local foliar salt-treatment model used in this study, electrophysiological signals recorded from the basal leaf region exhibited higher relative separability and clearer class boundaries. The basal region can therefore be regarded as the most favorable longitudinal recording region under the current experimental framework.

This result was broadly consistent with recent trends in plant electrophysiology research. Kozlova et al. pointed out that the plant electrome can integrate and reflect physiological changes across multiple levels, and that its interpretability depends strongly on recording quality and the analytical framework [1]. Barbosa-Caro and Wudick further noted that the generation and propagation of plant electrical signals involve coupled processes associated with membrane potential, Ca^2+^, pH, and reactive oxygen species, which suggests that changes in recording position may alter the observable surface electrical patterns [2]. Compared with these previous studies, the main contribution of the present work was not simply to further confirm that plant electrical signals can support recognition tasks. Rather, it was to show that even under the same plant, the same stimulus type, and the same recording system, longitudinal leaf position itself could substantially influence downstream recognition performance. This finding indicates that spatial position is an important upstream variable in intelligent analysis of plant electrophysiological signals, rather than a negligible detail of signal acquisition.

From a physiological perspective, the basal region is closer to the connection between the leaf and the main plant body, where vascular coupling is likely to be stronger. Local ion transport, changes in water status, and membrane-potential disturbances may therefore be projected more stably onto surface electrical signals in this region [1,2,3]. At the same time, the leaf surface is not an inert interface. Instead, it is closely associated with droplet retention, salt hygroscopicity and deliquescence, and local surface chemical processes [9,11]. Under local foliar salt treatment, different leaf regions may exhibit different response-coupling patterns because of differences in surface microstructure, wettability, and interface transport conditions. The superior classification performance of the basal region does not necessarily mean that its absolute physiological response is the strongest. However, it at least suggests that, within the current experimental framework, this region is more likely to generate temporal patterns that can be stably recognized by the model.

By contrast, the middle region may be more susceptible to the combined influence of local metabolic status and local environmental microfluctuations. Its electrophysiological signals may therefore reflect a stronger mixture of physiology-related variation and local random fluctuation. The apical region, as the distal part of the leaf, may be more likely to exhibit larger local fluctuations and greater interclass overlap under the current experimental framework. Previous studies have shown that the generation and propagation of plant electrical signals are jointly influenced by tissue status, ionic processes, and recording-interface conditions. In addition, differences in leaf-surface wettability, salt hygroscopicity, and local interface properties across leaf regions may further modify local uptake behavior and signal-response patterns [1,2,3,11,24]. It should be emphasized that this study did not directly measure local vascular density, ion flux, transpiration status, or water content in different leaf regions. Therefore, the interpretations above should be regarded as reasonable inferences based on existing studies of plant electrophysiology and leaf-surface processes rather than as mechanistic conclusions directly verified in this work. Based on the consistent results obtained across multiple models, the basal leaf region was selected as the optimal longitudinal region for subsequent hierarchy analysis.

### 2.3. Classification Results for Different Leaf Hierarchy Positions

**Figure 3:**
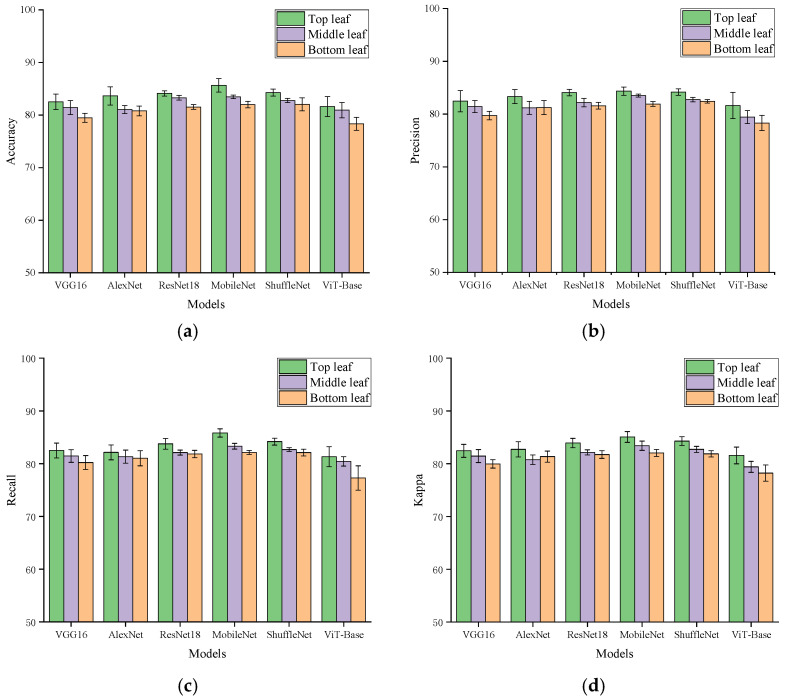
Comparison of multi-model classification performance across different leaf-level datasets within the optimal longitudinal region. (**a**) Accuracy; (**b**) Precision; (**c**) Recall; (**d**) Kappa. Error bars represent the mean ± standard deviation across five repeated runs.

After the basal leaf region was identified as the optimal longitudinal region, Figure 3 further showed that classification performance still differed substantially among the top, middle, and bottom leaves within this basal region. Among the three hierarchy levels, the basal region of the top leaf achieved the best overall average performance across all six models. This result indicated that, under the current experimental framework, the basal region of the top leaf was more suitable than the corresponding regions of the middle and bottom leaves for recording electrophysiological responses to local foliar salt treatment.

This finding suggests that, in addition to longitudinal leaf region, the vertical hierarchy of the leaf within the canopy also affected the discriminative quality of the recorded signals. Recent studies on plant wearables and leaf-surface monitoring have shown that recording quality and interpretability are jointly determined by the stability of the sensor–plant interface, the leaf-surface microenvironment, and local physiological activity [5,7]. Research in plant electrophysiology has also indicated that leaf responses to external stimuli are not distributed uniformly across the canopy. Instead, they are influenced by tissue developmental status, functional specialization, and the degree of local transport coupling [1,2,3]. In this context, the superior performance of the basal region of the top leaf in the classification task is physiologically plausible. Compared with lower leaves, upper functional leaves generally maintained higher tissue activity and stronger dynamic regulatory capacity, making them more likely to generate clear, stable, and discriminative temporal features under local stimulation.

Compared with previous studies, the contribution of this work is not simply to repeat the conclusion that plant electrical signals can distinguish different states. Rather, it further showed that different spatial leaf positions within the same plant do not provide equivalent information quality when responding to the same type of local stimulus. Recent studies on the plant electrome, wearable or implantable plant sensors, and physiological differences among leaf positions all suggest that signal acquisition outcomes are influenced not only by stimulus type but also by recording position, interface stability, and microenvironmental and physiological differences associated with leaf spatial location [1,4,25,26]. This implies that, when designing plant electrophysiological monitoring schemes, arbitrarily selecting a leaf as the recording target is insufficient. Both longitudinal leaf region and canopy hierarchy may affect the final recognition performance. It should be noted that this study did not directly quantify leaf age, transpiration intensity, metabolic activity, or differences in the leaf-surface microenvironment. Therefore, the basal region of the top leaf was better interpreted here as the optimal information source under the current experimental model and recording conditions rather than as a universal botanical rule. Through the stepwise optimization strategy of longitudinal region screening followed by hierarchy position screening, the basal region of the top leaf was ultimately identified as the optimal acquisition location for electrophysiological signals under local foliar salt treatment in *Clivia*.

### 2.4. Five-Gradient Recognition Results at the Optimal Location

**Figure 4:**
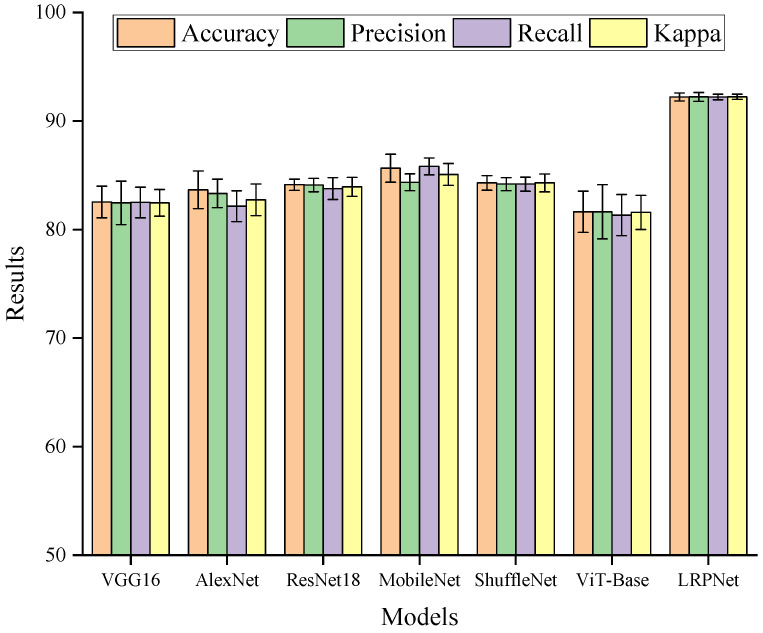
Comparison of the four evaluation metrics obtained by different models on the optimal dataset. Error bars represent the mean ± standard deviation across five repeated runs.

Based on the optimal acquisition location, namely the basal region of the top leaf, a five-gradient NaCl treatment recognition task was further constructed using 0, 100, 200, 300, and 400 mM to evaluate the performance of different models under a more complex classification scenario. As shown in Figure 4, all compared models exhibited a certain level of five-class recognition capability, but they differed substantially in both average performance and performance fluctuation. Under the current experimental setting, LRPNet achieved the highest mean values among all compared models for Accuracy, Precision, Recall, and Kappa, reaching 92.21%, 92.22%, 92.21%, and 92.23%, respectively.

These results indicated that acquisition-location optimization and model-structure optimization played complementary roles in the current task. The former ensured that the downstream classifier receives the most discriminative signal source available under the present experimental framework, whereas the latter further improved the efficiency of exploiting complex temporal patterns. For plant electrophysiological data, which are characterized by low amplitude, weak stability, and slow-varying trends, joint modeling of local details and global trends was necessary. In this respect, LRPNet achieved a better overall balance between the two.

Compared with previously published studies, the present results were consistent with the general conclusions of recent work on intelligent recognition of plant electrophysiological signals. Yao et al. used plant electrical signals in 2021 for salt-related deep learning prediction and recognition and showed that these signals contain long-term structures that can be learned by neural networks [12]. González I Juclà et al. achieved early recognition of nitrogen deficiency in tomato under greenhouse conditions using deep learning [13]. Yeom et al. and de Toledo et al. further showed that plant electrical signals can be used to distinguish changes in temperature, light, and water status, and that different modeling frameworks vary substantially in task suitability [14,15]. Compared with these studies, the main contribution of the present work is that the models were not compared directly using signals from an arbitrary acquisition location. Instead, the optimal spatial information source was first identified through systematic position screening, and the five-gradient recognition task was then conducted on that basis. This strategy placed model comparison on a more appropriate acquisition basis and more clearly revealed the relationship between recording location and model performance.

### 2.5. Ablation Experiment

To further verify the roles of the key modules in LRPNet, an ablation study was conducted on PoolFormer and ECA. Table 1 presents the mean results and standard deviations over 20 repeated runs. When PoolFormer was retained but ECA was removed, the model achieved Accuracy, Precision, Recall, and Kappa values of 87.28 ± 0.34%, 87.84 ± 0.26%, 87.30 ± 0.33%, and 88.19 ± 0.25%, respectively. When ECA was retained but PoolFormer was removed, these metrics increased to 89.81 ± 0.26%, 89.84 ± 0.37%, 89.95 ± 0.31%, and 89.94 ± 0.28%, respectively. When both modules were retained, the model performance further improved to 92.21 ± 0.37%, 92.22 ± 0.41%, 92.21 ± 0.26%, and 92.23 ± 0.24%, respectively.

These results indicate that the contributions of PoolFormer and ECA were complementary rather than redundant. The main role of ECA is to adaptively recalibrate different channels and thereby enhance more discriminative local patterns [27]. In contrast, PoolFormer mainly captures global trends and cross-temporal dependencies in the time-series structure [28]. For plant electrophysiological signals, which are characterized by low amplitude, high sensitivity to noise, local perturbations, and slow-varying global trends, the combination of these two mechanisms is more effective than either one alone [3,16,17]. In other words, ECA mainly helps the model emphasize which local channel responses deserve greater attention, whereas PoolFormer helps the model interpret local fluctuations within the broader temporal context. Their joint effect enables LRPNet to achieve the best mean performance in the multi-gradient recognition task.

From the perspective of structural design, the ablation results also suggested that the performance gain of the proposed network did not arise from simple module stacking. Instead, it resulted from the effective coordination between local feature enhancement and global information aggregation, which improved the discriminability of complex plant electrophysiological time series. This interpretation is also consistent with the observation that LRPNet achieved the highest mean performance in the model comparison.

### 2.6. Biological Significance and Methodological Boundaries

The main value of this study did not lie simply in showing that one network achieved higher classification metrics. More importantly, it demonstrated that the spatial acquisition location of plant electrophysiological signals can itself substantially affect downstream recognition performance. This finding was consistent with the general understanding emerging from recent studies of the plant electrome. Kozlova et al. pointed out that the plant electrome is not unstructured noise. Instead, it is closely associated with plant physiological status, abiotic stress tolerance, and rapid adaptive responses, and its monitoring value depends strongly on recording quality, analytical strategy, and the interpretive framework [1]. The present study further specified this idea at the acquisition level: even when the stimulus type, recording system, and modeling method were kept constant, the acquisition location itself remained an important upstream variable that determined the quality of information available to the model.

Based on the current results, this spatial heterogeneity may arise from the coupling of at least two types of factors. The first involves internal physiological and structural characteristics of the plant, including vascular connectivity, local ion transport, changes in water status, and membrane-potential propagation pathways. The second involves heterogeneity at the leaf-surface interface. Droplet retention, surface wettability, salt hygroscopicity and deliquescence, and foliar absorption processes may all influence the coupling between local treatment and local recording [9,11,24]. Previous studies have shown that different regions of the same leaf do not contribute equally to foliar uptake [24]. Therefore, under controlled local foliar salt treatment, differences in information quality across spatial positions may reflect not only differences in internal transport but also differences in leaf-surface interface processes.

At the same time, the boundaries of this study should be stated clearly. First, this study used a controlled local foliar salt-treatment model rather than a long-term root-zone salinization model. Therefore, the conclusions should not be directly extended to agricultural salinity mechanisms or recognition of long-term root-zone salt stress. Second, this study relied on sequential treatments and repeated within-plant measurements from only three plants. Because the number of independent biological replicates was limited, the results were more suitable for supporting relative comparisons than for supporting strong generalization claims. Third, the training and test sets were generated by random splitting of time-window samples, which means that data from the same plant and the same leaf could enter both subsets. Accordingly, the reported results should be interpreted as relative performance comparisons under a unified framework rather than as generalization estimates under strictly plant-independent conditions. Fourth, the interpretation of the advantages of the basal region and, in particular, the basal region of the top leaf was still mainly based on reasonable inference from existing studies of plant electrophysiology and leaf-surface processes. It has not yet been directly verified through physiological measurements such as local vascular structure, ion flux, transpiration status, leaf-surface wettability, or water content. Fifth, the Faraday cage in this study served only as part of the standardized noise-control environment rather than as an independently compared experimental variable. Therefore, no additional conclusion was drawn here regarding the effect of the shielded environment on physiological realism.

Despite these boundaries, this study still had clear methodological value. Previous studies have often fixed the acquisition location first and then directly compared model performance. In contrast, this study first demonstrated that spatial acquisition location itself was an upstream variable that should be explicitly optimized, and only then compared model performance at the optimal location. This forms a more complete research pathway: first optimize the signal source, and then optimize the classifier. This idea has broader implications for the design of intelligent plant electrophysiological monitoring systems. Model performance is determined not only by network architecture, but also by the upstream constraint imposed by acquisition location.

### 2.7. Future Research Directions

Based on the core hypothesis proposed and preliminarily supported in this study—that the spatial position of plant electrophysiological signals can significantly affect recognition performance under local salt stimulation—future research can proceed in several directions.

First, the reason why an “optimal location” emerges should be verified more rigorously at the physiological level. For example, future studies could combine measurements of local ion flux, water potential, transpiration rate, vascular bundle distribution, and histological characteristics, together with analyses of leaf-surface wettability and foliar absorption behavior [11,24], to establish a more complete evidence chain linking structure, interface, signal, and recognition.

Second, the effects of different preprocessing strategies on salt-treatment recognition should be examined more systematically. The current preprocessing pipeline, which uses wavelet denoising and sample-wise normalization, mainly emphasizes temporal pattern differences. However, the baseline shifts and absolute amplitude differences observed in the raw signals may also contain treatment-related information. Future work could compare multiple representation strategies, such as preserving only waveform-pattern features, preserving raw amplitude statistics, or combining waveform patterns with absolute amplitude information, to clarify the contribution of different feature sources to five-gradient recognition [12,13,14,15,16,17].

Third, more rigorous validation designs are needed to improve external validity. Specifically, future studies should adopt plant-independent or recording-session-independent data splitting strategies to test whether the ranking observed here, namely that the basal region outperforms the middle and apical regions and that the basal region of the top leaf outperforms the corresponding regions of other leaf hierarchies, still holds under datasets with higher independence. In addition, the transferability of this spatial optimization strategy should be examined across different plant species, different leaf morphologies, and different types of abiotic stress.

Fourth, the field should move from offline recognition toward real-time monitoring and device integration. Rapid advances in wearable and flexible plant sensors, as well as microneedle-based electrophysiological interfaces, indicate that stable, low-disturbance, and long-term recording is becoming increasingly feasible, while machine-learning-assisted analysis can further improve practical value [4,5,7]. Therefore, future work could build on the optimal site identified here, namely the basal region of the top leaf, and combine more stable flexible or wearable electrodes with lightweight model deployment to achieve continuous online monitoring of plant states.

## 3. Materials and Methods

### 3.1. Plant Materials and Growth Conditions

Mature potted *Clivia* plants were used in this study. *Clivia* was selected for three main reasons. First, its leaves are relatively thick, morphologically regular, and smooth on the surface, which facilitates stable application of surface electrodes and local salt gel. Second, its canopy has a clear hierarchical structure, which is suitable for spatial comparisons across leaf positions and leaf regions. Third, potted plants can be managed under relatively stable conditions, which helps reduce background environmental noise.

The experimental materials were obtained from the same commercial cultivation base. Three mature potted plants with uniform growth status and no visible pests or diseases were selected. Each plant had 15–17 healthy functional leaves, and the average plant height was approximately 30 cm. To minimize background variation among individuals, all plants were maintained under the same environmental conditions for at least 2 weeks before the experiment, with identical substrate, irrigation, and light management. The growth substrate consisted of humus soil, peat soil, and perlite mixed at a volume ratio of 2:1:1. During the experiment, soil moisture was maintained at approximately 25% through controlled irrigation to minimize interference from water fluctuations on leaf-surface electrical signals. Fertilization was stopped 1 week before the experiment to reduce background shifts caused by differences in nutritional status.

The plants were grown under a 12 h light/12 h dark photoperiod. Illumination was provided by full-spectrum LED plant growth lamps(LED grow light, E-Lite Semiconductor Co., Ltd., Chengdu, China), with red light (approximately 660 nm) and blue light (approximately 450 nm) as the main spectral bands, supplemented with a small amount of white light. The light intensity was controlled at 180–200 μmol m^−2^ s^−1^. During electrophysiological recording, the experiments were conducted in a constant-temperature and constant-humidity incubator. The air temperature was set at 20 °C and the relative humidity was maintained at 70% to reduce non-target signal variation caused by environmental fluctuations.

### 3.2. Construction of the Local Foliar Salt-Treatment Model

To establish a repeatable local salt-treatment model under controlled conditions, agarose gels containing different NaCl concentrations were used as the foliar treatment medium. Agarose was dissolved in deionized water to prepare a 1% (*w*/*v*) agarose solution. According to the experimental design, five NaCl concentration gradients were set: 0, 100, 200, 300, and 400 mM. Among them, 0 mM served as the salt-free control, 100 mM was used in the binary classification task for spatial position screening, and the remaining concentrations were used to extend the multi-gradient recognition task.

The gels were prepared as follows: Agarose powder was first weighed and added to deionized water, and the corresponding amount of NaCl was then added. The mixture was heated and stirred until fully dissolved. After the solution cooled to approximately 50–60 °C, it was poured into circular culture dishes with a diameter of 20 mm and a thickness of approximately 5 mm and allowed to solidify naturally, thereby forming agarose gels with different salt concentrations. The gels were then labeled, sealed, and stored for subsequent experiments.

This study did not attempt to fully simulate long-term root-zone salinization. Instead, it established a local foliar salt-treatment model that was spatially localized, repeatable, and convenient for comparison, in order to investigate differences in electrophysiological responses to local salt exposure across recording positions. Previous studies on leaf surfaces have shown that the leaf surface is closely coupled with atmospheric particles, salt deposition, liquid-film formation, and foliar absorption processes [9,10]. Therefore, although local foliar salt application cannot replace studies of long-term root-zone salt stress, it can provide a controlled experimental framework for analyzing the relationship between local foliar treatment and electrophysiological responses.

### 3.3. Electrophysiological Recording System

**Figure 5:**
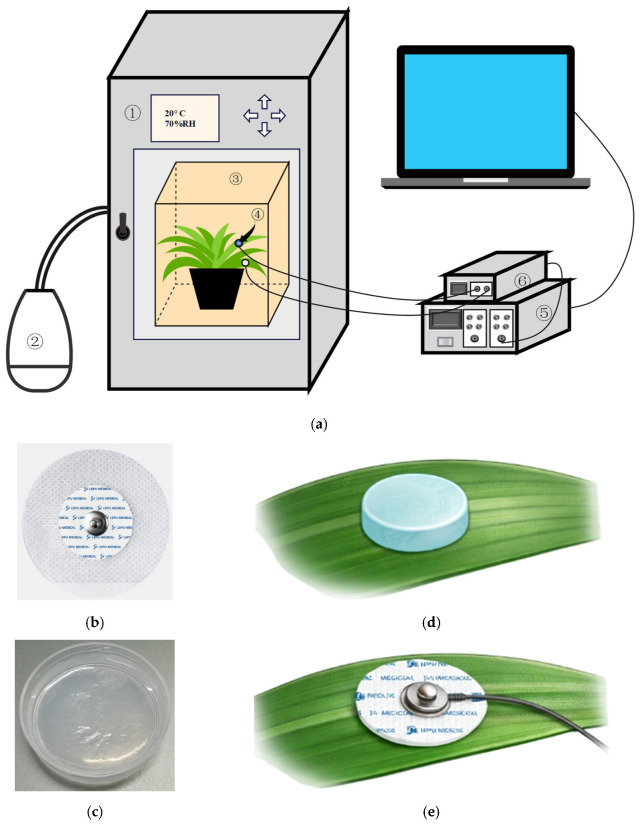
Experimental platform for electrophysiological recording under the controlled local leaf-surface salt-treatment model. (**a**) Schematic of the recording setup, including the Faraday cage and the temperature–humidity chamber; (**b**) surface recording electrode; (**c**) NaCl-containing agarose gel; (**d**) NaCl-containing agarose gel on the leaf surface; (**e**) surface recording electrode on the opposite side of the same leaf region.

To enable repeatable acquisition of low-amplitude electrical signals from the leaf surface, a plant electrophysiological recording platform was established. The platform consisted of surface electrodes, shielded wires, amplification and acquisition devices, and a constant-temperature and constant-humidity environment. The acquisition system is shown in Figure 5. Medical patch surface electrodes (TC63104, Qingdao Tenocom Medical Technology Co., Ltd., Qingdao, China) were used as recording electrodes and were attached to the abaxial side of the target leaf. The reference electrode was attached to the adaxial side of the lowest functional leaf of the plant to form a stable potential-difference measurement system. NaCl agarose gels with different concentrations were applied to designated regions on the adaxial side of the target leaf. The treatment region and the recording region were positioned to correspond as closely as possible within the same local spatial area, thereby improving consistency between local stimulation and local recording.

The acquired electrophysiological signals were transmitted through shielded wires (TC23106, Qingdao Tenocom Medical Technology Co., Ltd.) to a physiological signal acquisition system (RM6240CD, Chengdu Instrument Factory., Chengdu, China) for synchronous display and storage. Because plant leaf-surface electrical signals usually have low amplitudes, low signal-to-noise ratios, and high sensitivity to long-term drift, blackout cloth was used during the experiment to reduce interference from fluctuations in natural light. In addition, the entire recording system was placed inside a Faraday cage, and the cage was further placed inside a constant-temperature and constant-humidity incubator (HWS-80B, Tianjin Hongnuo Instrument Co., Ltd., Tianjin, China) to maintain stable temperature and humidity conditions.

It should be noted that the Faraday cage was used only as part of the standardized recording environment in this study to minimize uncontrollable electromagnetic fluctuations during signal acquisition. Because no parallel measurements were conducted outside the Faraday cage, this study does not independently evaluate whether shielding improved signal quality, nor does it draw a definitive conclusion regarding whether the shielding environment may have affected local plant physiological status. Therefore, the Faraday cage was treated only as part of the recording-condition control rather than as an experimental comparison variable.

### 3.4. Experimental Design

**Figure 6:**
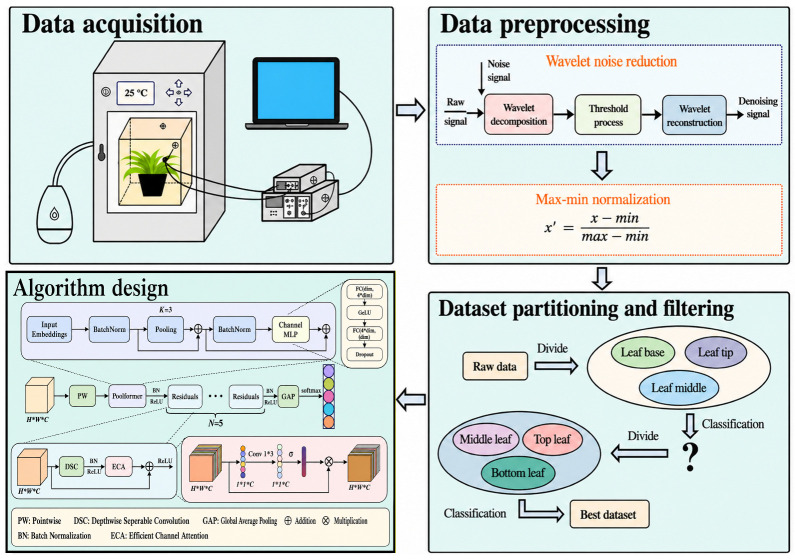
Overall flow of the proposed method. The framework consisted of three sequential stages: signal preprocessing, spatial sensitivity screening, and five-level classification on the optimal recording position. Spatial sensitivity screening included longitudinal-region comparison followed by leaf-level comparison.

Figure 6 illustrates the overall workflow of this study, which includes four components: data acquisition, data preprocessing, dataset construction and screening, and model design. The entire study was organized into three sequential stages: signal preprocessing, spatial sensitivity screening, and five-gradient recognition at the optimal location. The spatial sensitivity screening stage further included two steps: comparison of longitudinal leaf regions and comparison of leaf hierarchy positions.

#### 3.4.1. Definition of Leaf Spatial Regions and Leaf Hierarchy

**Figure 7:**
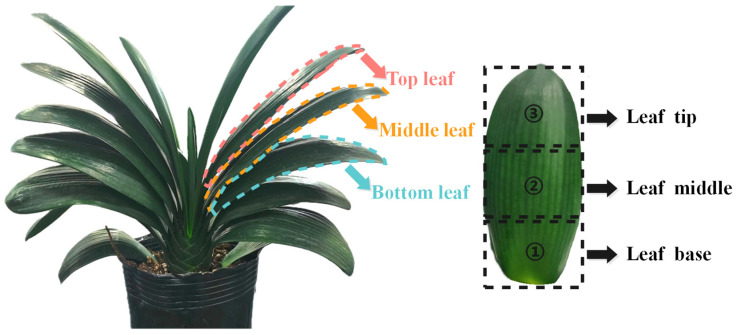
Schematic illustration of the spatial partitioning strategy used for leaf-position comparison in *Clivia*. Spatial positions were defined along two dimensions: longitudinal leaf region (leaf base, leaf middle, and leaf tip) and leaf level within the canopy (top leaf, middle leaf, and bottom leaf). The leaf-level categories were operationally defined from fully expanded, visually healthy functional leaves ranked by relative canopy height.

As shown in Figure 7, to systematically compare differences in responses to local salt treatment across spatial positions, the recording locations were defined along two dimensions: longitudinal leaf region and leaf hierarchy position.

For the leaf hierarchy dimension, a set of functional leaves eligible for comparison was first defined. Only fully expanded functional leaves without obvious mechanical damage, visible yellowing or senescence, abnormal coloration, or poor suitability for stable electrode attachment were included in the comparison. Newly emerged central leaves that had not fully expanded and outer leaves with clear aging symptoms were excluded. The selected functional leaves were then ranked according to their relative height within the canopy. One representative leaf was selected from the upper, middle, and lower thirds of the canopy and was operationally defined as the top, middle, and bottom leaf, respectively.

For the longitudinal dimension, each leaf was further divided into three regions: the basal region, middle region, and apical region. The middle region was defined as the midpoint along the leaf length. Based on this midpoint, the basal and apical recording regions were defined by extending 5 cm toward the leaf base and 5 cm toward the leaf tip, respectively. Through this combination, each plant provided nine potential recording locations: top/middle/bottom leaf × basal/middle/apical region.

#### 3.4.2. Spatial Sensitivity Screening Experiment

The purpose of the spatial sensitivity screening experiment was to compare the relative separability of different spatial locations under a unified classification task rather than to estimate their final generalization ability to entirely unseen plants. To achieve this, 0 mM and 100 mM NaCl agarose gels were applied separately at each candidate recording location, and electrophysiological signals were continuously recorded during treatment. The 0 mM condition served as the salt-free control, whereas the 100 mM condition served as the local salt-treatment condition for the binary classification task used in the screening stage.

Electrophysiological signal acquisition was performed in the following sequence. First, the solidified 0 mM and 100 mM NaCl agarose gels were removed from the culture dishes and applied sequentially to the basal region of the bottom leaf of the first *Clivia* plant. Each treatment lasted 2 h, during which electrophysiological signals were continuously recorded. The same procedure was then repeated at the middle and apical regions of the same leaf. After all nine candidate positions of the first plant had been measured, the remaining two *Clivia* plants were tested in the same order to ensure procedural consistency.

A 0.5 h resting interval was introduced between different salt treatments. The same 0.5 h interval was also used when the recording region was changed. The main purpose of this design was to reduce the immediate residual influence of the preceding treatment on the subsequent one while keeping the electrode arrangement, local tissue status, and contact impedance as consistent as possible. Previous studies have shown that plant electrical responses usually involve both rapid signal changes and slower subsequent physiological adjustment processes [1,2,29]. Therefore, the 0.5 h recovery interval should be regarded as an operational compromise. It helps reduce immediate carry-over effects between adjacent treatments, but it cannot fully exclude the influence of slower recovery processes. Accordingly, the present sequential treatment design should be regarded as an experimental framework for comparing relative response differences under a unified recording interface, rather than as a strictly randomized biological validation design capable of fully separating treatment effects from order effects.

#### 3.4.3. Five-Gradient Extended Recognition Experiment

After stepwise screening identified the optimal recording location, a five-gradient NaCl treatment recognition experiment was further conducted at this location using three *Clivia* plants. In addition to 0 mM and 100 mM, 200, 300, and 400 mM NaCl treatments were included, resulting in a five-class recognition task. The purpose of this stage was to verify whether the proposed LRPNet could extract stable and discriminative features from more complex multi-gradient electrophysiological time series when signals were recorded at the optimal spatial location.

### 3.5. Dataset Construction

(1) Individual Variation and Data Integration Principle

Different *Clivia* plants may differ in baseline physiological status, leaf developmental stage, and response amplitude to local salt treatment. However, the goal of spatial position screening in this study was neither to establish a plant-specific model nor to estimate inter-plant response variation. Instead, the goal was to identify leaf spatial acquisition locations that exhibit relatively consistent sensitivity across plants. Therefore, during dataset construction, recordings with the same spatial attributes from different plants were integrated to extract response patterns that were relatively stable across individuals. This integration strategy does not imply that individual variation was ignored. Rather, such variation was treated as background variability in the spatial sensitivity analysis so that the relative separability of different spatial positions could be compared under a unified evaluation framework.

(2) Sampling Parameters and Sample Construction Principle

All electrophysiological signals were recorded at a sampling frequency of 30 Hz. Because plant leaf-surface electrical signals are mainly characterized by low-frequency, slowly varying, and nonstationary fluctuations, a sampling rate of 30 Hz was sufficient to capture the major temporal dynamics required for the current task while avoiding redundant data accumulation associated with excessively high sampling rates. For sample construction, each independent time-window sample consisted of a continuous 30 s signal segment containing 900 data points. A 30 s window was selected because this duration could capture a relatively complete potential-fluctuation process under local foliar treatment while still producing a sufficient number of samples for model training. This design therefore balances feature representation and training efficiency.

(3) Construction of the Longitudinal Position Sensitivity Dataset

The longitudinal position sensitivity analysis was designed to compare the relative separability of the basal, middle, and apical leaf regions under local salt treatment. Specifically, under 0 mM and 100 mM NaCl conditions, electrophysiological signals were recorded from the basal, middle, and apical regions of nine leaves included in the analysis across three *Clivia* plants. At each recording position, signals were continuously recorded for 2 h under each treatment. After segmentation with fixed 30 s windows, 240 samples were obtained for each plant–leaf–position–concentration condition.

During the data integration stage, samples from the same longitudinal region across the three *Clivia* plants were merged. All basal-region samples were combined to form the basal-region binary classification dataset, all middle-region samples were combined to form the middle-region binary classification dataset, and all apical-region samples were combined to form the apical-region binary classification dataset. For any given longitudinal region, the total number of samples was calculated as follows: 3 plants × 3 leaves × 2 concentrations × 240 time windows = 4320 samples. Therefore, the three longitudinal-region datasets contained 12,960 samples in total.

(4) Construction of the Leaf Hierarchy Sensitivity Dataset

The leaf hierarchy sensitivity analysis was not conducted in parallel with the longitudinal analysis. Instead, it was performed after the comparison of longitudinal leaf regions had been completed. Specifically, the binary classification performance of the basal-, middle-, and apical-region datasets was first compared to identify the longitudinal region showing the most prominent response to local salt treatment. This region was then defined as the optimal longitudinal region. Based on this result, response differences among different leaf hierarchy positions, namely the top leaf, middle leaf, and bottom leaf, were further analyzed within this optimal longitudinal region to determine the final optimal spatial acquisition location.

In the hierarchy analysis stage, the optimal longitudinal region was fixed as a common spatial constraint, and the corresponding data from the top, middle, and bottom leaves of the three *Clivia* plants were reorganized to construct three binary classification datasets. Each hierarchy level corresponded to one representative leaf per plant. Therefore, the total number of samples for each hierarchy dataset was calculated as follows: 3 plants × 1 representative leaf per plant × 2 concentrations × 240 time windows = 1440 samples. Accordingly, the three hierarchy datasets contained 4320 samples in total.

The logic of this design is that, at the hierarchy comparison stage, the longitudinal region had already been determined through the previous screening process. Therefore, samples from the same hierarchy level and the same optimal longitudinal region across different plants could jointly represent the overall response characteristics of that hierarchy level under the current experimental model. By comparing the classification performance of these three hierarchy datasets, the most sensitive leaf hierarchy within the optimal longitudinal region could be further identified, thereby forming a stepwise optimization strategy of longitudinal region screening followed by hierarchy position screening.

(5) Construction of the Five-Gradient Dataset at the Optimal Acquisition Location

After the two-stage screening of longitudinal leaf region and leaf hierarchy position, the optimal acquisition location under the current experimental framework was determined. To further validate the ability of electrophysiological signals recorded at this location to distinguish different treatment levels and to provide a data basis for subsequent multiclass modeling, a five-gradient NaCl treatment experiment was conducted at the optimal acquisition location using concentrations of 0, 100, 200, 300, and 400 mM.

In the five-gradient experiment, these five salt concentrations were applied at the optimal acquisition location in each of the three *Clivia* plants, and electrophysiological signals were continuously recorded for 2 h under each concentration. Consistent with the previous dataset construction procedure, the sampling frequency was set to 30 Hz and samples were segmented using fixed 30 s windows. Thus, under each plant–position–concentration condition, 240 samples were obtained. Because the optimal acquisition location corresponded to one recording position in each of the three plants, the number of samples in each salt-concentration class was 3 plants × 240 time windows = 720 samples. Therefore, the five concentration classes yielded a total of 3600 samples.

(6) Dataset Splitting Strategy and Inference Boundary

To ensure consistency in the evaluation framework across different tasks, the longitudinal position sensitivity datasets, the leaf hierarchy sensitivity datasets, and the five-gradient dataset at the optimal acquisition location were all divided into training and test sets using a random split based on time-window samples. Specifically, each dataset was randomly divided at a ratio of 80%/20%, with 80% used for training and 20% used for testing.

For each binary classification dataset in the longitudinal position sensitivity analysis, the total number of samples was 4320, including 3456 training samples and 864 test samples. For each binary classification dataset in the leaf hierarchy sensitivity analysis, the total number of samples was 1440, including 1152 training samples and 288 test samples. For the five-class dataset at the optimal acquisition location, the total number of samples was 3600, including 2880 training samples and 720 test samples. Under class-balanced conditions, each concentration class contributed 576 samples to the training set and 144 samples to the test set.

It should be noted that the time-window samples were segmented from 2 h continuous raw recordings obtained from the same plant, the same leaf, and the same treatment condition using a 30 s window length. Therefore, the training and test sets were not fully independent at the plant, leaf, or recording-session level. In other words, although the same 30 s time-window sample did not appear in both the training and test sets, different time-window samples derived from the same plant, the same leaf, or the same 2 h continuous recording could still be assigned separately to the training and test sets. Because of this characteristic, the classification results reported in this study are mainly intended for comparing the relative performance of different spatial locations and different models under a unified dataset construction framework. Their absolute values should not be interpreted directly as estimates of generalization performance under strictly plant-independent conditions. For the present study, the main advantage of this splitting strategy is that it enables more efficient use of limited data while allowing for fair comparison across longitudinal regions, hierarchy positions, and model structures under a consistent standard.

It should also be emphasized that the three *Clivia* plants used in this study are better regarded as biological material sources, whereas the continuous recordings collected under different positions and treatments mainly constitute repeated measurements within plants. In other words, the focus of this study is relative spatial sensitivity analysis under a controlled comparison framework rather than strong generalization estimation based on a large number of independent plants. Therefore, the reported results are more suitable for comparing the relative performance of different spatial positions and different models under a unified experimental framework and should not be directly extrapolated as absolute generalization performance under strictly plant-independent conditions.

### 3.6. Data Preprocessing

**Figure 8:**
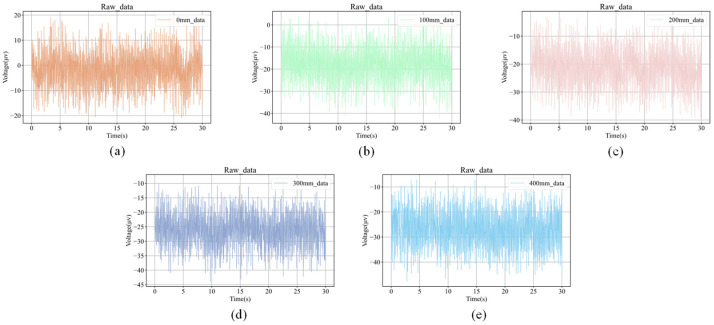
Representative electrophysiological signal samples of *Clivia* under five NaCl treatment levels. (**a**) 0 mM NaCl; (**b**) 100 mM NaCl; (**c**) 200 mM NaCl; (**d**) 300 mM NaCl; (**e**) 400 mM NaCl.

As shown in Figure 8, plant leaf-surface electrical signals are characterized by low frequency, low amplitude, and nonstationarity, and the raw recordings are often accompanied by high-frequency random noise, baseline fluctuations, and environmental interference [1,3,8,12,16,17]. Therefore, before model training, the raw signals were processed sequentially using wavelet threshold denoising and min–max normalization.

**Figure 9:**
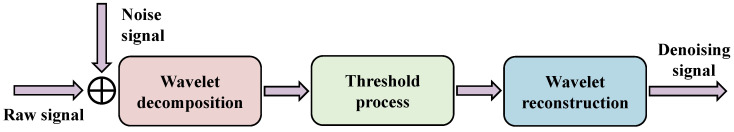
Schematic illustration of the wavelet-based denoising procedure.

Wavelet-threshold denoising was used because it could effectively suppress random noise in frequency bands close to those of the target signal while preserving the main temporal structure and local waveform characteristics, making it suitable for weakly nonstationary signals such as plant electrophysiological recordings [16,17]. Normalization was used to reduce differences in absolute amplitude scale among samples and to improve numerical stability and convergence efficiency during network training. The principle of wavelet denoising is illustrated in Figure 9, and the normalization formula is given in Equation (1).

(1) $$x^{\prime }=\frac{x-x_{\min}}{x_{\max}-x_{\min}}$$

where *x* denotes the raw data point, *x_min_* and *x_max_* denote the minimum and maximum values within the sample, respectively, and *x*′ denotes the normalized result.

It should be noted that sample-wise normalization improves the comparability of waveform patterns across different time windows, but it also compresses differences in absolute potential level and baseline offset in the raw signals. Therefore, the preprocessing framework used in this study places greater emphasis on characterizing temporal structure and relative fluctuation patterns under different treatment conditions, while preserving relatively less information about absolute potential magnitude. Accordingly, the subsequent classification analysis mainly reflects the separability of normalized electrophysiological temporal patterns rather than discrimination based primarily on raw absolute potential values.

### 3.7. Model Design

**Figure 10:**
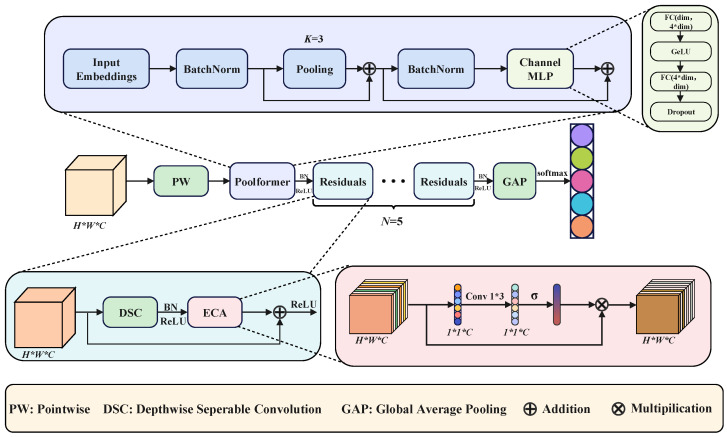
Architecture of the proposed LRPNet model. The network consisted of a pointwise convolution embedding layer, a PoolFormer block, enhanced residual blocks, global average pooling, and a Softmax classifier. PW denotes pointwise convolution, DSC denotes depthwise separable convolution, and GAP denotes global average pooling.

Under limited-sample conditions, a plant electrophysiological recognition model should satisfy three requirements. First, it should extract informative local features from low-amplitude and weakly stable time series. Second, it should capture cross-temporal dependencies that may arise during local foliar treatment. Third, its complexity should remain low enough to support future deployment in embedded or portable systems. Based on these considerations, LRPNet, a lightweight residual network integrated with the PoolFormer architecture, was proposed. The model architecture is shown in Figure 10, and the model parameters are listed in Table 2.

The input to LRPNet was a preprocessed 1 × 30 × 30 sample matrix. The network mainly consists of a pointwise convolution embedding layer, a PoolFormer module, five enhanced residual blocks, and a global average pooling layer followed by a Softmax classifier. Compared with a purely convolutional structure, this design maintains a relatively small parameter scale while balancing local detail extraction, cross-temporal information aggregation, and channel-wise feature recalibration.

(1) Pointwise Convolution Embedding:

Pointwise convolution uses a 1 × 1 convolution to perform channel mapping and feature expansion without changing the spatial resolution. For plant electrophysiological time-series matrices, in which feature differences are often subtle, pointwise convolution can re-encode the input channels at low computational cost and allow subsequent modules to extract discriminative patterns in a higher-dimensional representation space. Compared with directly using large convolution kernels or stacking standard convolutions, pointwise convolution helps reduce the number of parameters and improves the efficiency of early feature representation.

(2) PoolFormer Module:

Recent advances in vision Transformers have shown that global information modeling can improve complex pattern recognition performance [23]. However, standard self-attention structures are sensitive to sample size and computational cost. Based on the MetaFormer architecture, PoolFormer replaces complex self-attention operations with a lighter token-mixing mechanism, thereby retaining global representation capability while substantially reducing computational burden [28]. In this study, PoolFormer was introduced after the pointwise convolution layer to enhance the network’s ability to capture overall temporal trends and cross-temporal dependencies. For plant electrophysiological signals, which are characterized by low amplitude and pronounced slow-varying trends, this type of lightweight global information aggregation was considered appropriate.

(3) Enhanced Residual Blocks and the ECA Mechanism:

Residual connections can alleviate gradient vanishing in deep network training and preserve low-level feature information [20]. Because plant electrophysiological signals contain many weak but important local variations, depthwise separable convolution was adopted in the residual blocks to improve local feature extraction efficiency while controlling parameter size [21,22]. In addition, to strengthen effective interactions among channels, the Efficient Channel Attention (ECA) mechanism was incorporated into the residual blocks to adaptively enhance more discriminative channel responses [27]. ECA performs feature recalibration through lightweight local cross-channel interaction, which makes it well suited for lightweight network design under limited-sample conditions.

(4) Output Layer:

After feature extraction, the network uses Global Average Pooling (GAP) to aggregate the spatial features of each channel into a single statistical representation, which is then fed into a Softmax classifier to produce class probabilities. Compared with a fully connected layer, GAP can substantially reduce the number of parameters and the risk of overfitting. This design was better suited to plant electrophysiological tasks, in which the sample size was limited but clear interclass discrimination remained important.

### 3.8. Training Settings and Evaluation Metrics

All models were trained using the Adam optimizer [18]. To balance convergence speed and training stability, the training hyperparameters were determined by grid search. The final settings were as follows: an initial learning rate of 1 × 10^−4^, a batch size of 30, and 200 training epochs. To reduce fluctuations caused by random initialization, all compared models were independently run five times under the same data split, training strategy, and hyperparameter settings. The mean and standard deviation of Accuracy, Precision, Recall, and Kappa were reported as the final results. This design was intended to evaluate the average performance and stability of different models under a unified experimental setting and to compare their relative performance within the current experimental framework.

## 4. Conclusions

Under a controlled local foliar salt-treatment model, this study systematically investigated the spatial optimization of electrophysiological signal acquisition in *Clivia* leaves. The results showed that plant electrophysiological signals exhibited pronounced spatial heterogeneity. In the comparison of longitudinal leaf regions, the basal region consistently showed higher average classification performance than the middle and apical regions. In the comparison of leaf hierarchy positions, the basal region of the top leaf further exhibited the best overall discriminative capability.

Based on this optimal location, a five-gradient NaCl treatment recognition task was constructed using 0, 100, 200, 300, and 400 mM, and the lightweight residual network LRPNet was proposed. The results showed that LRPNet achieved the highest mean performance among the compared models in terms of Accuracy, Precision, Recall, and Kappa, with an Accuracy of 92.21%. The ablation study further indicated that PoolFormer and ECA played complementary roles in global-trend modeling and local discriminative feature enhancement, and that their joint use improved recognition of complex plant electrophysiological time series.

More importantly, this study showed that spatial acquisition location was not a secondary experimental condition in plant electrophysiological monitoring. Instead, it was an important upstream variable that affected both the quality of the information available to the model and the final recognition performance. This study provided a unified methodological framework for acquisition-location optimization and lightweight recognition in plant electrophysiology. However, the current conclusions mainly apply to the controlled local foliar salt-treatment model established here. More rigorous physiological validation, cross-species testing, and higher-independence data partitioning are still needed to further evaluate the broader applicability of this framework.

## Figures and Tables

**Table 1:** Classification results of the ablation analysis.

Model	Poolformer	ECA	Accuracy (%)	Precision (%)	Recall (%)	Kappa (%)
1	√	×	87.28 ± 0.34	87.84 ± 0.26	87.30 ± 0.33	88.19 ± 0.25
2	×	√	89.81 ± 0.26	89.84 ± 0.37	89.95 ± 0.31	89.94 ± 0.28
3	√	√	92.21 ± 0.37	92.22 ± 0.41	92.21 ± 0.26	92.23 ± 0.24

**Table 2:** Layer configuration of the proposed LRPNet model.

Input	Layer	Filter Size	Filter Number	Stride
1 × 30 × 30	PW conv	1 × 1	16	1
16 × 30 × 30	Poolformer + BN + ReLU	-	-	-
16 × 30 × 30	Residuals + BN + ReLU	3 × 3	32	1
32 × 30 × 30	Residuals + BN + ReLU	3 × 3	64	1
64 × 30 × 30	Residuals + BN + ReLU	3 × 3	96	1
96 × 30 × 30	Residuals + BN + ReLU	3 × 3	128	1
128 × 30 × 30	Residuals + BN + ReLU	3 × 3	128	1
128 × 30 × 30	GAP	-	-	-
128 × 1 × 1	Softmax	-	-	-

## Data Availability

Data will be made available on request.
